# The Reliability and Validity of Quality of Life Questionnaire Upper Limb Lymphedema (ULL-27) Turkish Patient With Breast Cancer Related Lymphedema

**DOI:** 10.3389/fonc.2020.00455

**Published:** 2020-05-12

**Authors:** Ayşe Kayali Vatansever, Tuğba Yavuzşen, Didem Karadibak

**Affiliations:** ^1^Bayrakli District Health Directorate, Izmir, Turkey; ^2^Institute for Oncology, Dokuz Eylul University, Izmir, Turkey; ^3^Physiotherapy and Rehabilitation, Dokuz Eylul University, Izmir, Turkey

**Keywords:** ULL-27 quality of life questionnaire, breast cancer, lymphedema, quality of life, eortc30

## Abstract

**Purpose:** Breast cancer is the most common cancer amongst women both in Turkey and in the world. Lymphedema, which negatively affects the quality of life, is one of the most prevalent problems reported by breast cancer survivors. Upper Limb Lymphedama 27 (*ULL-27*) questionnaire is a valid and reliable tool that assesses the quality of life in patients with breast cancer-related lymphedema. Until now, a Turkish-language version was lacking. The aim of this study was to perform a cross-cultural validation and reliability of the Turkish version of the ULL-27 questionnaire.

**Methods:** This cross-sectional study involved forward- backward translation, and cross-cultural adaptation. 81 women (mean age and body mass index 54.96 ± 11.35 years and 29.50 ± 5.74 kg/m^2^) who had breast cancer related-upper extremity lymphedema were evaluated using the ULL-27 Quality of life questionnaire-Turkish version. Assessment of limb size was quantified by using circumferential limb measurements. European Organization for Research and Treatment of Cancer (EORTC) 30-item Quality of Life Questionnaire and Quality of Life Questionnaire breast cancer-23 (QLQ-BR23) were analyzed by Pearson's correlation analysis with the ULL-27 Turkish Version to indicate the convergent validity. Cronbach's alpha (internal consistency) and exploratory factor analysis were used to assess the questionnaire's reliability.

**Results:** The mean of lymphedema duration and severity were 23.12 ± 30.88 months. Mild lymphedema was reported in 42% (34 people) of the cases included in the study. It was observed that 33.3% (27 people) had moderate lymphedema and 24.7% (20 people) had severe lymphedema. The alpha coefficient (internal consistency) for the Turkish *ULL-27* total score was high (alpha = 0.93). Content validity was good because all questions were understandable for all participants (The alpha coefficient for the subgroups of the scale of physical, psychological, social scores, were 0.90, 0.87, and 0.75, respectively). External construct validity was highly confirmed by expected correlations with comparator scales, EORTC-30, and QLQ-BR23 (*p* < 0.01).

**Conclusions:** The Turkish version of the ULL-27 Questionnaire is a valid and reliable tool for evaluating QoL in women with upper limb lymphedema related to breast cancer.

## Introduction

Breast cancer is still the most common type of cancer among women in the world (1). Its incidence rates have been increasing mostly in developing countries, including Turkey (2). But breast cancer survival rates have also increased worldwide. The recent decline in breast cancer mortality in many countries might be due to early diagnosis and improved treatment protocols (3, 4). Among the many symptoms, lymphedema is one of the most common side effects of breast cancer treatment.

A recent meta-analysis of women with breast cancer, the lymphedema rate was 21.4%. The risk of developing lymphedema is especially high during the first two years of the surgery (5). Many sources indicate the likelihood of lymphedema development between 2 and 50% (6–9). Lymphedema is a chronic and progressive condition resulting from an abnormality of, or damage to, the lymphatic system. Any reduction in the capacity of the lymphatic system to drain fluid from the interstitium and return it to the blood circulation will cause fluid to build up in the skin and subcutaneous tissues of the affected part of the body. It is known to negatively affect the quality of life (QoL) in breast cancer survivors due to limb swelling, heaviness, pain, pitting of skin, tightness or hardness in the limb, inflammation, and reduced mobility in the shoulder and arm (10–14).

There is a widespread awareness among researchers on the importance of assessing the specific quality of life related to lymphedema. On the other hand, very few specific questionnaires have been developed on upper extremity lymphedema. Upper Limb Lymphedema 27 (ULL-27), introduced by Launois et al. (15) is a scale that can describe all symptoms in one form, can provide a holistic approach, is easy to use, and can evaluate their ability to perform common functional activities in patients with Breast Cancer Related Lymphedema (BCRL). However, The ULL-27 has been validated in very few countries. Therefore, the aims of this study were: (1) to perform a translation and cross-cultural adaptation of the ULL-27 among patients with breast cancer related-upper extremity lymphedema, to investigate the scale's validity, and to conduct exploratory factor analysis (confirmatory factor analysis has been done previously in other languages) with responsiveness within a Turkish-speaking population sample; and (2) to assess quality of life in Turkish patients with breast cancer related-upper extremity lymphedema.

## Materials and Methods

### Study Design and Participants

This study was performed on 81 women who had developed upper extremity lymphedema after breast cancer treatment. Participants who were referred to Dokuz Eylul University (DEU) Hospital, Department of Medical Oncology in Izmir, Turkey between June 2016 and May 2017 were assessed in the School of Physical Therapy. All participants were informed about the purpose and the procedures of the study and signed an informed consent form according to guidelines approved by the university hospital ethical committee. Ethical protocol number was 2543-GOA and decision number was 2016/07-23.

To meet the inclusion criteria, patients had to: (a) be aged 18 and over; (b) have received no local and systemic treatment (colorectal surgery, chemotherapy, radiotherapy) in the last 6 months; (c) able to read, write, and understand Turkish; (d) have mild–moderate-severe degreed lymphedema; (e) be willing and able to attend the study. Women were ruled ineligible according to the following exclusion criteria: malignant lymphedema; recurrent cancer or infection in the arms; severe disorders related to cognition, muscles, or joints.

### Assessment

A complete medical history was obtained from each participant, including demographic information (i.e., age, gender, height, weight, body mass index [BMI], occupation, dominant hand, and affected hand) and disease characteristics (i.e., type and side). In addition, the type of operation, the number of excised lymph nodes, radiotherapy session received, other treatments, lymphedema duration, and previous infection attacks were also recorded.

### Circumferential Measurement

Edema was assessed by circumferential measurement (CM). CM were taken with participants in a supine position and the arm abducted at 30^o^C. The circumference of both limbs was measured every 5 cm, starting at the nail bottom of 3rd fingers and continuing 50 cm proximally. The difference between both arms were recorded in cm. All patients were evaluated with the same standard tape measure (150 cm length, 7 mm width). The severity of the edema was done according to the criteria set by the American Physical Therapy Association. According to this, the difference between both limbs is slightly less than 3 cm, the middle 3–5 cm, anything over 5 cm was recorded as severe lymphedema (16).

### Design

This cross-sectional methodological study involved translation, back translation, and cross-cultural adaptation, that is, localization. To assess the questionnaire's reliability, Cronbach's alpha (for internal consistency) and exploratory factor analysis were conducted. To indicate the convergent validity, Pearson's correlation analysis was performed with the European Organization for Research and Treatment of Cancer Quality of Life Cancer module (EORTC QLQ-C30) and the European Organization for Research and Treatment of Cancer Quality of Life—Breast Cancer Module (EORTC BR-23) for which reliability and validity studies have been conducted in the Turkish-speaking population.

Women participating in the research were evaluated by the same researchers; information was given about the purpose and methods of the study. All measurements were carried out face to face with the participants. All evaluations lasted about 45-60 min.

### Quality of Life

EORTC QLQ-C30, EORTC BR-23 and ULL-27 were used to measure QoL.

The EORTC QLQ-C30 is composed of 30 items assessing global perceived health status and QoL (QL2). These items are grouped into five functional scales (physical-PF2, role-RF2, cognitive-CF, emotional-EF, and social functioning-SF); three symptom scales (fatigue-FA, nausea & vomiting-NV, and pain-FA); six single item scales—dyspnea-DY, insomnia-SL, appetite loss-AP, constipation-CO, diarrhea-DI, and financial difficulties-FI (17).

QLQ-BR23 has 23 items to assess functional scales (Body Image-BRBI, Sexual Functioning-BRSEF, Sexual Enjoyment-BRSEE, and Future Perspective-BRFU); symptom scales (systemic therapy side effects -BRST, breast symptoms -BRBS, arm symptoms -BRAS, and upset by hair loss-BRHL) (17).

The QLQ scores vary from 0 (worst) to 100 (best) for the functional and global health status (GHS) parameters and from 0 (best) to 100 (worst) for symptoms parameters. A five-point difference in QoL scores is considered the minimum clinically significant difference. Both questionnaires were cross-culturally adapted to Turkish by Demirci et al. (18).

The original ULL-27 was created by Launois et al. (15). It is a questionnaire that evaluates the quality of life in three dimensions in subjects with upper limb lymphedema. The scale consists of 27 questions with physical, psychological, and social dimensions. 5-point Likert scoring scale (1 = strongly disagree, 5 = strongly agree) is used. The first 15 questions are on the physical dimension (min 15 and max 75 points), the questions between 16 and 22 on social dimension (min 7 and max 35 points), and the questions between 23 and 27 evaluate the social dimension (min 5 and max 25 points) of the individual. The total score of 27 questions is calculated for the global score. The lowest score is 27 and the highest score is 135 points. The high score of the scale shows that it affects the quality of life of the individual badly (15).

### Convergent Validity

Convergent validity is the principle that measures theoretically similar constructs that should be highly intercorrelated. The convergent validity of two similar constructs can be estimated using correlation coefficients. To test the hypothesis for convergent validity for the ULL-27, we used the EORTC QLQ-C30, QLQ-BR23. Convergent validity in subjects with upper extremity lymphedema after breast cancer treatment was evaluated by investigating correlations between the scale's psychometric parameters and the commonly used assessments EORTC QLQ-C30, QLQ-BR23.

### Translational and Cross-Cultural Adaptation

The process of translation and cross-cultural adaptation, that is, localization, was carried out according to Beaton's guidelines (19, 20).

(a) Translation into Turkish: the ULL-27 was translated from English into Turkish in accordance with Newmark's concept of “communicative translation” to achieve a dynamic equivalence between the source and target texts. “Communicative translation attempts to produce in its readers an effect as close as possible to that obtained on the readers of the original.” The text was independently translated by two native Turkish speakers, one of whom was a linguist and the other a health care professional who knew English as a second language. Finally, both target texts were compared for equivalent effect, and a single version was agreed upon. (b) Back translation into English: two bilingual translators with English as a first language back translated the agreed Turkish version into English taking into account cultural adaptation, that is, the localization process. They compared the two versions and agreed on a single version. (c) Review committee: the final version was submitted to a bilingual committee consisting of clinicians and translators. The text was checked for semantic and idiomatic equivalence acceptable for dynamic equivalence. Step 3 ended with a final approval. (d) Test of the prefinal version: the prefinal version was sent to the authors of the original form, and their comments were taken into consideration. Then, the final version was piloted with 15 women by testing what was meant by each item and response chosen in order to verify whether the formulation of the item was clear or not. All of the findings were reevaluated by the expert committee. Finally, the back translation of the scale was approved by the author who composed the original form.

### Statistical Analysis

Data analysis was made with Statistical Package for Social Science (SPSS) version 20. All categorical data frequency and percentage were calculated. Descriptive statistics on the demographics of patients were used to show information about cancer and lymphedema. Statistical significance level was regarded as 0.05 for all tests. Confirmatory factor analysis was used to test the construct validity of the questionnaire. In light of the assumptions set forth in the multiple regression analysis to examine a dependent variable, Path analysis was performed on all arguments. Quality of life survey to measure the reliability and Cronbach's alpha coefficients were calculated to measure the internal consistency. Cronbach's alpha was determined to be an acceptable level of reliability above 0.7. A poll of the *Kolmogorov-Smirnov test* to measure compliance that conforms to a normal distribution was made. Three major scores of the questionnaire (physical, psychological, and social) and the correlation between the content in question was examined by *Spearman correlation* test. ULL27 life-selected EORTC QLQ-C30 and QLQ-BR23 questionnaires quality of parallel survey evaluated concurrent validity by calculating the *Pearson correlation*.

### Ethical Considerations

The study was approved by the local University Medical Ethics Committee, and the patients gave their written informed consent to take part in the research prior to the study. R. Launois, the creator of the original ULL-27, was asked for permission to apply the scale in a convergent validity study for the Turkish language. In addition, during the ethical considerations, the Head of the Dokuz Eylul University Faculty of Medicine, Department of Oncology, approved the study to be held in their department.

## Results

Patients' compliance during evaluation was good. The EORT-C30 and BR23 questionnaires were handed out to the patients and they were requested to fill in the forms. Eighty-one patients diagnosed BCRL with a mean age of 54.96 ± 11.35 years were enrolled in the study. Demographic and clinical data related to the patients are given in Table 1.

**Table 1:** Demographic characteristics of the participants (*n* = 81).

Age (years) (X ± SD)	54.96 ± 11.35
BMI (kg/m^2^) (X ± SD)	29.50 ± 5.74
Waist circumference (cm) (X ± SD)	95.39 ± 10.50
Hip circumference (cm) (X ± SD)	109.73 ± 10.34
Occupation (%)	
Housewife	59.3
Worker	24.7
Retired	16
Dominant arm (%)	
Right	92.6
Left	7.4
Effected arm (%)	
Right	43.2
Left	56.8
First observed part of lymphedema in arm (%)	
Hand	21
Forearm	19.8
Upper arm	29.6
Severity of lymphedema (%)	
Mild	42
Moderate	33.3
Severe	24.7
Type of Operation (%)	
Lumpectomy	47
Total mastectomy	53
Treatments (%)	
ET+CT+RT	41.98
CT+RT	44.44
RT	13.58
Lymph nodes removed (number) (X ± SD)	15.94 ± 8.36
History of recurrent lymphangitis (%)	
Yes	24.7
No	75.3
Duration of lymphedema (months) (X ± SD)	23.12 ± 30.88

*SD, Standard Deviation; BMI, Body Mass Index; ET, endocrine therapy; CT, chemotherapy; RT, radiotherapy*.

### Reliability of ULL-27 Questionnaire

The reliability of the scale, internal consistency, and item scores were investigated in terms of correlation and invariance. ULL-27 internal consistency of the quality of life questionnaire (reliability) was assessed by Cronbach's alpha score. Analysis of the internal consistency of all cases related to the scale of its response to the ULL-27 quality of life questionnaire was out of the total score. Croncbach alpha coefficient of *0.93* was found. Subgroups of the scale of physical scores had an alpha coefficient of *0.90*, psychological *0.87*, and social score *0.75* identified. Accordingly, the survey revealed that the degree of internal consistency was good. According to this model, when we look at the reliability analysis, all questions are consistent and valid for the Turkish people, without removing any items from the original survey (Table 2). Agent scale correlation (inter correlation) was rated on the same answer and substance-test are displayed by calculating the correlation coefficient. The obtained substance-test coefficients of correlation *r* = 0.43 *and r* = 0.87 was found to take values from Table 3. Test-retest analysis scale was determined by inter class correlation method. The test was applied twice to the last 15 patients at 20-day intervals. In both applications, the reliability coefficient taken according to the total scores was *r* = 0.40 (*p* > 0.05). Calculating a consistency coefficient, item-correlation coefficients and the number of test-retest times were used to show statistical significance. These results scale internal consistency, substance-test shows that the correlation coefficient is high and test-retest reliability examined for the test.

**Table 2:** ULL-27 quality of life questionnaire of physical, psychological, social, and global measures of reliability test.

	**Cronbach's alpha**	**Number of questions**
Physical Score	0.90	15
Psychological Score	0.87	7
Social Score	0.75	5
Global Score	0.93	27

**Table 3:** Reliability of each question in ULL-27.

	**Mean ± SD**	**α**	***r***
Difficulties grasping high objects	3.17 ± 1.31	0.90	0.47
Difficulties maintaining certain positions	3.07 ± 1.33	0.90	0.46
Arm feels heavy	3.30 ± 1.38	0.89	0.69
Arm feels swollen	3.54 ± 1.30	0.90	0.57
Difficulties getting dressed	2.72 ± 1.32	0.89	0.74
Having trouble getting to sleep	2.74 ± 1.38	0.90	0.45
Having trouble sleeping	2.81 ± 1.33	0.90	0.50
Difficulties grasping objects	2.75 ± 1.26	0.89	0.68
Difficulties holding objects	2.96 ± 1.35	0.90	0.64
Difficulties walking heavy arm	2.53 ± 1.33	0.89	0.77
Difficulties washing	2.32 ± 1.30	0.89	0.73
Difficulties taking public transport	2.49 ± 1.34	0.89	0.68
Tingling, burning feelings	2.67 ± 1.36	0.90	0.43
Feelings of swollen, hard, tense skin	3.15 ± 1.33	0.90	0.53
Difficulties in working relationship and tasks	2.75 ± 1.19	0.90	0.60
Feeling sad	2.61 ± 1.25	0.73	0.73
Feeling discouraged	2.41 ± 1.25	0.87	0.87
Feeling lack of self-confidence	2.45 ± 1.27	0.67	0.67
Feeling distressed	2.79 ± 1.22	0.81	0.81
Feeling well in oneself	2.82 ± 1.14	0.26	0.26
Feeling a wish to be angry	2.50 ± 1.25	0.54	0.54
Having confidence in the future	2.61 ± 1.30	0.74	0.74
Difficulties taking advantage of good weather, in life outside the house	2.36 ± 1.31	0.55	0.55
Difficulty with personal projects holidays and hobbies	2.89 ± 1.32	0.60	0.60
Difficulties in emotional life with spouse or partner	2.33 ± 1.11	0.53	0.53
Difficulty in social life	2.63 ± 1.13	0.55	0.55
Fearful of looking in a mirror	1.52 ± 0.838	0.34	0.34

### ULL-27 Validity of Questionnaire

**Figure 1:**
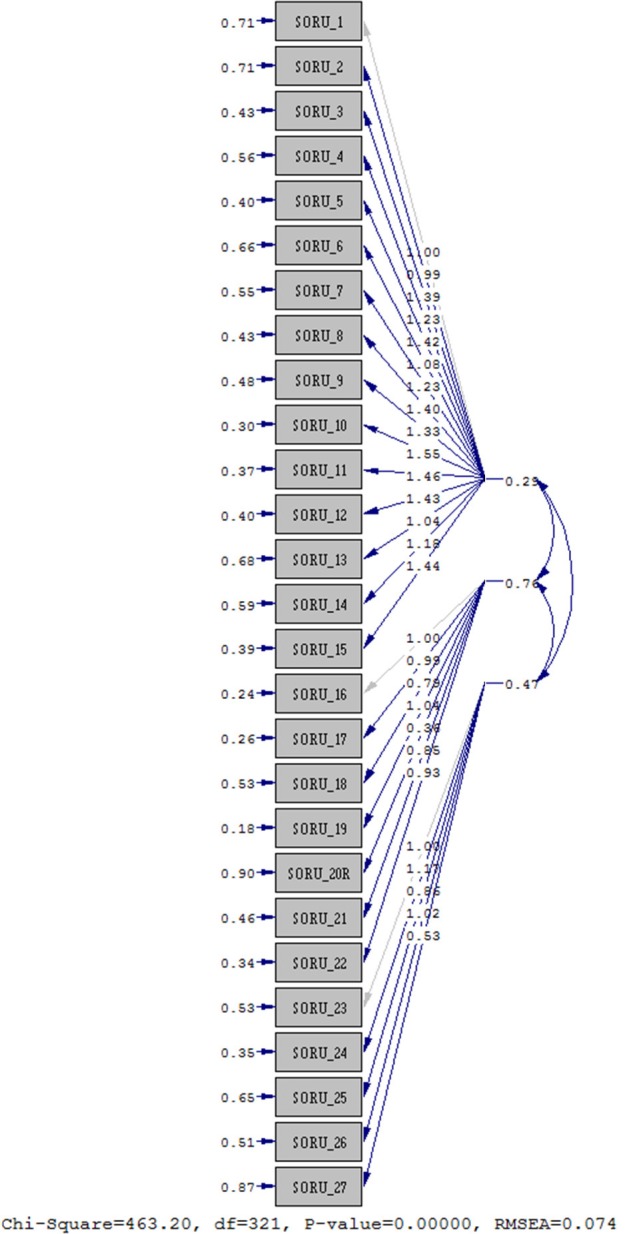
Path diagram.

The validity of the ULL-27; parallel forms (concurrent) were analyzed in two ways: validity and construct validity. ULL-27 was used in order to determine the construct validity of the questionnaire survey according to the applied confirmatory factor analysis. It was first seen in the value of RMSEA confirmatory factor analysis. The RMSEA value of our study was found to be 0.074. According to the Path diagram, the first 15 questions were on the physical score in the Turkish version, which gives the item distribution as in the original form of the UL-27 quality of life questionnaire. Questions between 16 and 22 give the psychological score and questions between 23-27 give the social score (*x*^2^ = 463.20) (*p* = 0.000) (Table 4, Figure 1). Whether the relationship between the variables-assumed absence model that the difference *Comperative Fit Index (CFI)* according to close to the minimum *(0.97)* and *Incremental Fit Index (IFI)* based on “acceptable harmony” *(0.97)* was detected. Goodness of Fit Index measured the sample covariance matrix of the model *(GFI)*, what is viewed as “acceptable harmony” was determined to be in the group (0.96). With the ULL-27, scoring a minimum of 0 (27) and a maximum of 100 (135) points formula used to be;(*total score*−*minscore*)/(*max score*−*min score*) *x*100

**Table 4:** ULL-27 Quality of Life questionnaire indices of confirmatory factor analysis.

**Index**	**RMSEA**	**CFI**	**IFI**	**GFI**
ULL-27 Life Quality Questionnaire	0.074	0.97	0.97	0.96

*RMSEA Index (Root Mean Square Error of Approximation), CFI (Comperative Fit Index) and IFI (Incremental Fit Index)*.

**Graph 1:**
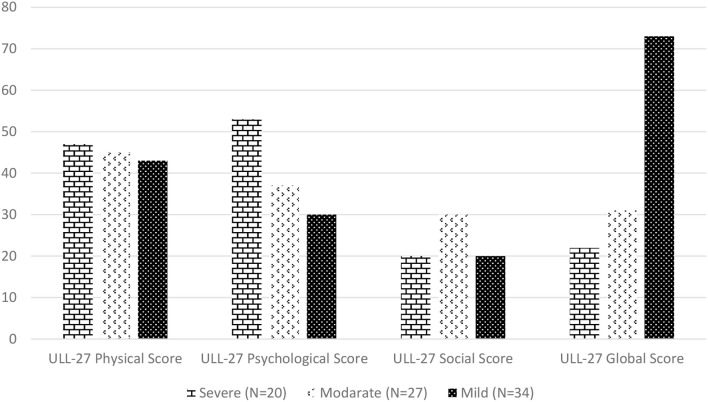
According to lymphedema severity quality of life scores of ULL-27.

Accordingly, 81 individuals participated in the study and the ULL-27 global score for quality of life was found to be 42.54 ± 19.71 (Table 5). ULL-27 quality of life questionnaire of the *Kolmogorov-Smirnov and Shapiro-Wilk test* was performed to examine whether they fit a normal distribution. The test does not conform to a normal distribution (*p* < 0.05). ULL-27 questionnaire of physical, psychological, social, and global relationship between the score and the questions were analyzed with Pearson's correlation coefficient. It was found to be statistically significant in itself (*p* < 0.01) (*p* < 0.05). With the Turkish version of ULL-27 quality of life questionnaire, a parallel score was found within the scope of EORTC-QLQ-C30 and BR23 related validity. The correlation between the vertex of all cases of this survey were analyzed by Spearman correlation coefficient. Accordingly, the global score of the ULL-27 questionnaire and the correlation coefficient between BR23 and C30 and the scores of the scale were found to be significantly similar (*p* < 0.05) (Table 5). A statistically significant difference was found between the psychological items of the ULL-27 questionnaire between diarrhea, sexual function, sexual pleasure, breast symptoms, and sadness that caused hair loss (*p* < 0.05). Shortly, we found that symptom scores worsened as the severity of lymphedema increased. Accordingly, we saw that the quality of life decreased (Graph 1). We have demonstrated that the Turkish version of the ULL-27 quality of life questionnaire we evaluated was a valid and reliable test battery for the use of patients to evaluate the condition.

**Table 5:** Correlations between ULL-27 and EORT QLQ C30; BR-23 parameters.

	**Physical score (46.64 ± 21.90)**	**Psychological score (40.12 ± 23.65)**	**Social score (33.64 ± 20.52)**	**Global score (42.54 ± 19.71)**
QL2	−0.208	−0.472**	−0.324**	−0.337**
(60.79 ± 18.93)	0.062	**0.000**	**0.003**	**0.002**
PF2	−0.564**	−0.333**	−0.459**	−0.546**
(62.88 ± 21.51)	**0.000**	**0.002**	**0.000**	**0.000**
RF2	−0.410**	−0.349**	−0.449**	−0.437**
(63.87 ± 31.94)	**0.000**	**0.001**	**0.000**	**0.000**
EF	−0.177	−0.526**	−0.362**	−0.328**
(70.23 ± 24.88)	0.115	**0.000**	**0.001**	**0.003**
CF	−0.101	−0.323**	−0.269*	−0.204
(69.95 ± 24.91)	0.372	**0.003**	**0.015**	0.068
SF	−0.151	−0.266*	−0.297**	−0.221*
(75.71 ± 26.88)	0.178	**0.016**	**0.007**	**0.047**
FA	0.362**	0.356**	0.421**	0.417**
(44.57 ± 26.43)	**0.001**	**0.001**	**0.000**	**0.000**
NV	0.347**	0.364**	0.406**	0.369**
(12.75 ± 24.33)	**0.001**	**0.001**	**0.000**	**0.000**
PA	0.450**	0.417**	0.519**	0.503**
(40.32 ± 28.11)	**0.000**	**0.000**	**0.000**	**0.000**
DY	0.384**	0.297**	0.343**	0.403**
(23.24 ± 29.77)	**0.000**	**0.007**	**0.002**	**0.000**
SL	0.398**	0.465**	0.488**	0.467**
(37.72 ± 33.58)	**0.000**	**0.000**	**0.000**	**0.000**
AP	0.266*	0.402**	0.379**	0.355**
(8.64 ± 18.08)	**0.016**	**0.000**	**0.000**	**0.001**
CO	0.081	0.244*	0.170	0.149
(24.48 ± 31.07)	0.474	**0.028**	0.130	0.186
DI	0.147	0.212	0.221*	0.207
(11.52 ± 25.90)	0.189	0.057	**0.047**	0.064
FI	0.122	0.273*	0.161	0.177
(22.83 ± 29.39)	0.277	**0.014**	0.152	0.113
BRBI	−0.183	−0.321**	−0.239*	−0.248*
(71.19 ± 25.10)	0.102	**0.004**	**0.032**	**0.025**
BRSEF	0.065	0.019	0.081	0.068
(81.89 ± 24.60)	0.566	0.866	0.472	0.545
BRSEE	0.045	0.015	0.088	0.049
(78.59 ± 30.41)	0.688	0.891	0.433	0.662
BRFU	−0.241*	−0.423**	−0.351**	−0.343**
(48.13 ± 30.27)	**0.030**	**0.000**	**0.001**	**0.002**
BRST	0.268*	0.381**	0.389**	0.348**
(28.59 ± 18.12)	**0.015**	**0.000**	**0.000**	**0.001**
BRBS	0.228*	0.202	0.215	0.225*
(29.82 ± 24.11)	**0.041**	0.071	0.054	0.044
BRAS	0.606**	0.313**	0.504**	0.562**
(49.84 ± 25.52)	0.000	0.004	0.000	0.000
BRHL	0.176	0.064	0.012	0.122
(15.21 ± 28.87)	0.115	0.569	0.918	0.278

** *p < 0.001*,

* *p < 0.005*.

*physical-PF2, role-RF2, cognitive-CF, emotional-EF, social functioning-SF, fatigue-FA, nausea & vomiting-NV, pain-FA, dyspnea-DY, insomnia-SL, appetite loss-AP, constipation-CO, diarrhea-DI, financial difficulties-FI, Body Image-BRBI, Sexual Functioning-BRSEF, Sexual Enjoyment-BRSEE, Future Perspective-BRFU, systemic therapy side effects -BRST, breast symptoms -BRBS, arm symptoms –BRAS, upset by hair loss-BRHL*.

*The values in the table show that the ones with negative sign are negative meaningful and those without sign have positive meaning*.

## Discussion

After breast cancer treatment approaches, people face several problems. These problems affect the quality of life of the individual. Lymphedema is one of these problems. Therefore, it is important to measure the degree to which the quality of life in people with BCRL is affected. In our study, we assessed reliability and validity of the Turkish version of ULL-27 in the upper limb lymphedema. The ULL-27 was found to be a valid and reliable measure in Turkish patients with BCRL. Previous studies stated that upper limb lymphedema effects patient's lives in different ways and there were many symptoms which were specific like heaviness and swollen limbs. The SF-36, EORTC QLQ C-30, and BR-23 are the most preferable scales for patients having upper limb lymphedema. Lymphedema specific questionnaires such as Lymph ICF, LyQLI, and LYMQOL have started to be used to assess patients by professionals (21–23). EORT QLQ C-30 and BR-23 are the most frequently used parameters to assess the disease-specific quality of life in people who have had breast cancer in Turkey. So, EORT QLQ C-30, BR-23, and ULL-27 was the main assessment parameters in this study. The EORT QLQ C-30 involved all breast cancer symptoms, and only four out of nine specific questions were about arm symptoms (18, 24, 25). Its manual scoring take long time, whereas ULL-27 is only about upper limb lymphedema symptoms and quickly calculates the score. Pusic et al. showed that according to COSMIN criteria the ULL-27 was the only scale that could be used with patients that left no doubt on the results (18). ULL-27 physical, psychological, and social scores of Cronbach's alpha values were supported by Launois and Viehoff. Launois et al. were calculated in the same way (15). Similarly, Viehoff et al. reported that the Dutch version of the questionnaire was a valid and reliable study, Cronbach's alpha values were found to be close (26). Our values showed parallel values. In this study we found physical, psychological, and social Cronbach's alpha values that were relatively high. Global score Croncbach's alpha was found by calculating the high reliability of the questionnaire. ULL-27 and EORT QLQ C-30, BR-23 sub parameters were found to be highly correlated. In order to be able to compare our results with those of the original questionnaire, the tests were performed in a similar attitude. A factor analysis was done with RMSEA, Comparative Fit Index (CFI), and Incremental Fit Index (IFI). Goodness of Fit Index (GFI) was evaluated for covariance and we found that ULL-27 questionnaire is suitable for Turkish BCRL patients. Structural equation of the questionnaire showed high adaptation.

Physical scores of individuals (ULL-27) was found to be an average. We considered this a score that increases an individual's quality of life deteriorated. The EORTC C30 is consistent with the scores of the quality of life, physical function, role function, emotional function, cognitive function and social function parameters and the physical score of the ULL 27 quality of life questionnaire. The higher the ULL-27 quality of life score, the higher the other parameters. According to the analysis of ULL-27 in individuals with high physical score points, we saw a low score of the role and function scale in EORT C-30. We have seen that pain, weakness, nausea-vomiting, insomnia, dyspnea, anorexia, constipation, diarrhea, and financial parameters decrease the quality of life. We found that when individuals' ULL-27 physical score increased, fatigue, nausea, vomiting, pain, shortness of breath, insomnia, and loss of appetite worsened. We have also seen that high ULL-27 physical score has a negative impact on BR-23 body image, sexual function, sexual satisfaction, and future opinion parameters. Likewise, we found that patients with the highest physical score had higher breast and arm symptoms.

Psychological and social dimensions also affect individuals' quality of life. The high points of sexual function and sexual pleasure have a negative impact on an individual's quality of life. Our study is high in these two parameters. We observed that the psychological score of ULL-27 worsened as the hair loss symptom score increased. One of the side effects of chemotherapy is hair loss. Although time has passed, this causes us to think that the effect of this situation continues. In this study, we found that the physical and cosmetic effects of treatments generally affect the social and psychological state of those with BCRL.

One study limitation was that there were not enough participants. This lack of participants might have affected the results of our study. One strength of our work was that all patients were women. Lymphedema after breast cancer in women is very high so we think that our results are close to the general population.

In conclusion, the ULL-27 questionnaire seems to be a reliable and valid scale for assessing the quality of life in Turkish upper limb lymphedema patients. It is available for use in clinical practice and research.

## Data Availability Statement

The raw data supporting the conclusions of this article will be made available by the authors, without undue reservation.

## Ethics Statement

This article does not contain any studies involving animals performed by any of the authors. All procedures performed in studies involving human participants were in accordance with the ethical standards of the institutional research committee and with the 1964 Helsinki Declaration and its later amendments or comparable ethical standards. Informed consent was obtained from all individual participants involved in the study.

## Author's Note

The ULL27 was developed by Professor Robert Launois with an educational grant from REES France. Any person who wishes to use the questionnaire should contact Professor Robert Launois (reesfrance@wanadoo.fr).

## Author Contributions

AK and DK conceived of the presented idea. TY developed the theory and performed the computations. AK and DK verified the analytical methods. DK encouraged AK to investigate (a specific aspect) and supervised the findings of this work. All authors discussed the results and contributed to the final manuscript.

## Conflict of Interest

The authors declare that the research was conducted in the absence of any commercial or financial relationships that could be construed as a potential conflict of interest.

## References

[1] Ghoncheh M Pournamdar Z Salehiniya H. Incidence and mortality and epidemiology of breast cancer in the world. Asian Pacific J Cancer Prev. (2016) 17:43–6. 10.7314/APJCP.2016.17.S3.43 27165206

[2] Ozmen V Ozmen T Dogru V. Breast cancer in turkey; an analysis of 20.000 patients with breast cancer. Eur J Breast Heal. (2019) 15:141–6. 10.5152/ejbh.2019.4890 31312788 PMC6619786

[3] Rivera-Franco MM Leon-Rodriguez E. Delays in breast cancer detection and treatment in developing countries. Breast Cancer Basic Clin Res. (2018) 12:117822341775267. 10.1177/1178223417752677 29434475 PMC5802601

[4] Narod SA Iqbal J Miller AB Why have breast cancer mortality rates declined? J Cancer Policy. (2015) 5:8–17. 10.1016/j.jcpo.2015.03.002

[5] DiSipio T Rye S Newman B Hayes S. Incidence of unilateral arm lymphoedema after breast cancer: A systematic review and meta-analysis. Lancet Oncol. (2013) 14:500–15. 10.1016/S1470-2045(13)70076-7 23540561

[6] Soojin Ahn Elisa Port R Mount Sinai Medical Center. Lymphedema precautions: time to abandon old practices? J Clin Oncol. (2016) 34:691. 10.1200/JCO.2015.64.9574 26712226

[7] Huang T-W Tseng S-H Lin CC Bai CH Chen CS Hung CS. Effects of manual lymphatic drainage on breast cancer-related lymphedema: a systematic review and meta-analysis of randomized controlled trials. World J Surg Oncol. (2013) 11:15. 10.1186/1477-7819-11-15 23347817 PMC3562193

[8] Tsai RJ Dennis LK Lynch CF Snetselaar LG Zamba GK Scott-Conner C. The risk of developing arm lymphedema among breast cancer survivors: A meta-analysis of treatment factors. Ann Surg Oncol. (2009) 16:1959–72. 10.1245/s10434-009-0452-2 19365624

[9] Warren LEG Miller CL Horick N Skolny MN Jammallo LS Sadek BT. The impact of radiation therapy on the risk of lymphedema after treatment for breast cancer: A prospective cohort study. Int J Radiat Oncol Biol Phys. (2014) 88:565–71. 10.1016/j.ijrobp.2013.11.232 24411624 PMC3928974

[10] Shaitelman SF Radiation therapy targets and the risk of breast cancer-related lymphedema: a systematic review and network meta-analysis. Breast Cancer Res Treat. (2016) 162:201–15. 10.1007/s10549-016-4089-0 28012086

[11] Schmitz KH Ahmed RL Troxel A Cheville A Smith R Lewis-Grant L Bryan CJ. Weight lifting in women with breast-cancer-related lymphedema. N Engl J Med. (2009) 361:664–73. 10.1056/NEJMoa0810118 19675330

[12] Norman SA Localio AR Potashnik SL Cheville A Smith R Lewis-Grant L Lymphedema in breast cancer survivors: incidence, degree, time course, treatment, and symptoms. J Clin Oncol. (2009) 361:664–73. 10.1200/JCO.2008.17.9291 PMC2645852 19064976

[13] Schmitz KH Troxel AB Cheville A Grant LL Bryan CJ Gross CR. Physical activity and lymphedema (the PAL trial): Assessing the safety of progressive strength training in breast cancer survivors. Contemp Clin Trials. (2009) 30:233–45. 10.1016/j.cct.2009.01.001 19171204 PMC2730488

[14] Wanchai A Armer JM Stewart BR Lasinski BB Breast cancer-related lymphedema: A literature review for clinical practice. Int J Nurs Sci. (2016) 3:207–9. 10.1016/j.ijnss.2016.04.006

[15] Launois R Mègnigbêto AC Pocquet K Alliot F A specific quality of life scale in upper limb lymphedema : the ULL-27 questionnaire. Genoa Lymphol. (2001) 35:1–760. 10.1016/S1098-3015(11)71503-0

[16] Warren AG Brorson H Borud LJ Slavin SA. Lymphedema: A comprehensive review. Ann Plast Surg. (2007) 59:464–72. 10.1097/01.sap.0000257149.42922.7e 17901744

[17] Aaronson NK Ahmedzai S Bergman B Bullinger M Cull A Duez NJ. The European Organization for Research and Treatment of Cancer QLQ-C30: a quality-of-life instrument for use in international clinical trials in oncology. J Natl Cancer Inst. (1993) 85:365–76. 10.1093/jnci/85.5.365 8433390

[18] Demirci S Eser E Ozsaran Z Tankisi D Aras AB Ozaydemir G. Validation of the Turkish versions of EORTC QLQ-C30 and BR23 modules in breast cancer patients. Asian Pac J Cancer Prev. (2011) 12:1283–7. 21875283

[19] Bombardier C Guillemin F Ferraz M Recommendations for the cross-cultural adaptation of health status measures. Am Acad Orthop Surg. (1998) 1–27.

[20] Beaton DE Bombardier C Guillemin F Ferraz MB. Guidelines for the process of cross-cultural adaptation of self-report measures. Spine. (2000) 25:3186–91. 10.1097/00007632-200012150-00014 11124735

[21] Devoogdt N Van Kampen M Geraerts I Coremans T Christiaens MR. Lymphoedema functioning, disability and health questionnaire. (Lymph-ICF) reliability and validity. Phys Ther. (2011) 91:944–57. 10.2522/ptj.20100087 21493748

[22] Klernäs P Johnsson A Horstmann V Kristjanson LJ Johansson K. Lymphedema quality of life inventory. (LyQLI). -development and investigation of validity and reliability. Qual Life Res. (2015) 24:427–39. 10.1007/s11136-014-0783-8 25633655

[23] Keeley V Crooks S Locke J Veigas D Riches K Hilliam R A quality of life measure for limb lymphoedema (LYMQOL). J Lymphoedema. (2010) 5:26–37.

[24] Kim EJ Ko SK Kang HY. Mapping the cancer-specific EORTC QLQ-C30 and EORTC QLQ-BR23 to the generic EQ-5D in metastatic breast cancer patients. Qual Life Res. (2012) 21:1193–203. 10.1007/s11136-011-0037-y 22012023

[25] Leng T Ching S Idris D Loh SY Seow GC Chia YY Validation of EORTC QLQ-C30 and QLQ-BR23 questionnaires in the measurement of quality of life of breast cancer patients in Singapore. Asia-Pacific J Oncol Nurs. (2014) 1:22–32. 10.4103/2347-5625.135817 PMC5123455 27981079

[26] Viehoff PB van Genderen FR Wittink H. Upper limb lymphedema 27. (ULL27). : dutch translation and validation of an illness-specific health-related quality of life questionnaire for patients with upper limb lymphedema. Lymphology. (2008) 41:131–8. 19013881

